# Red meat, processed meat, and other dietary protein sources and risk of overall and cause-specific mortality in The Netherlands Cohort Study

**DOI:** 10.1007/s10654-019-00483-9

**Published:** 2019-01-23

**Authors:** Piet A. van den Brandt

**Affiliations:** 1 Department of Epidemiology, GROW-School for Oncology and Developmental Biology, Maastricht University Medical Centre, P.O. Box 616, 6200 MD Maastricht, The Netherlands; 2 Department of Epidemiology, CAPHRI-School for Public Health and Primary Care, Maastricht University Medical Centre, Maastricht, The Netherlands

**Keywords:** Processed meat, Red meat, Protein sources, Mortality, Neoplasms, Cardiovascular diseases, Respiratory diseases, Cohort studies

## Abstract

Processed meat and red meat have been associated with increased mortality, but studies are inconsistent and few have investigated substitution by other protein sources. The relationship of overall and causes-specific mortality with red meat, processed meat, and other dietary protein sources was investigated in The Netherlands Cohort Study. In 1986, 120,852 men and women aged 55–69 years provided information on dietary and lifestyle habits. Mortality follow-up until 1996 consisted of linkage to statistics Netherlands. Multivariable case-cohort analyses were based on 8823 deaths and 3202 subcohort members with complete data on diet and confounders. Red meat (unprocessed) intake was not associated with overall and cause-specific mortality. Processed meat intake was significantly positively related to overall mortality: HR (95% CI) comparing highest versus lowest quintile, 1.21 (1.02–1.44) with P_trend_ = 0.049. Significant associations were observed for cardiovascular [HR Q5 vs. Q1, 1.26 (1.01–1.26)] and respiratory [HR = 1.79 (1.19–2.67)], but not cancer mortality [HR = 1.16 (0.97–1.39)]. Adjustment for nitrite intake attenuated these associations which became nonsignificant: HRs Q5 versus Q1 (95% CI) were: 1.10 (0.77–1.55) for total, 1.09 (0.71–1.67) for cardiovascular, 1.44 (0.68–3.05) for respiratory, and 1.11 (0.78–1.58) for cancer mortality. Nitrite was significantly associated with overall, CVD and respiratory mortality. Poultry intake was significantly inversely related to cancer and overall mortality. While fish intake showed positive associations, nut intake showed inverse associations with all endpoints. Replacing processed meat with a combination of poultry, eggs, fish, pulses, nuts and low-fat dairy was associated with lower risks of overall, cardiovascular and respiratory mortality. Processed meat was related to increased overall, CVD and respiratory mortality, potentially due to nitrite. Substituting processed meat with other protein sources was associated with lower mortality risks.

**Electronic supplementary material:**

The online version of this article (10.1007/s10654-019-00483-9) contains supplementary material, which is available to authorized users.

## Introduction

Processed meat and unprocessed red meat intake have been linked to overall and cause-specific mortality in prospective studies, but the evidence for unprocessed red meat intake is not consistent. A recent meta-analysis on red and processed meat and mortality [1] found a difference in direction of associations with red meat between cohort studies from the United States (relative risk, comparing high vs. low intake, 1.23, 95% CI 1.17–1.30), from Asia (RR = 0.93, 95% CI 0.88–0.99) and from Europe (RR = 0.90, 95% CI 0.59–1.38), although only two studies from Europe were available [2, 3].

For processed meat, most cohort studies found positive associations with mortality, but the evidence regarding cause-specific mortality is less consistent. Most often, only cardiovascular and cancer mortality were specifically studied in this respect [1]. Furthermore, little work has been done on respiratory mortality and processed meat intake which were recently found to be related [4]. Individual types of processed meat have rarely been studied with regard to mortality, as well as the relation with nitrite intake, an important preservative used in processed meat.

Apart from red and processed meat, other dietary protein sources have been studied to varying extent with regard to mortality (e.g., [5–17]). These sources include poultry, eggs, fish, legumes, nuts, and dairy foods. While meta-analyses for nuts show a consistently inverse association [14], a recent meta-analysis on fish [12] shows large variations in associations between fish consumption and mortality between cohorts from Europe versus other continents. Again, only few studies from Europe were available [18, 19].

To further investigate the associations of unprocessed red meat, processed meat, and other dietary protein sources with risk of overall and cause-specific mortality, data from a large European cohort study, The Netherlands Cohort Study, were used. In addition, the effect of substituting other protein sources for processed meat on mortality was examined.

## Methods

### Study design and mortality follow-up

The NLCS started in September 1986 and includes 58,279 men and 62,573 women aged 55–69 years [20]. At baseline (September 1986), they completed a mailed, self-administered 11-page questionnaire on cancer risk factors. The NLCS study was approved by the Maastricht University institutional review board. For efficiency, we applied the nested case-cohort method [21], requiring only data-entry of questionnaires (which could not be scanned) of cases and a random subcohort. Following this method [20], cases were enumerated from the entire NLCS-cohort of 120,852 (numerator information of mortality rates), whereas the accumulated person-years at risk in the cohort were estimated using a subcohort of 5000 subjects (denominator information). The case-cohort method implies that the persontime at risk is estimated through a sample of the total cohort, instead of actively following the total cohort. Data entry of questionnaires is only needed for cases and subcohort members, instead of the total cohort [20, 21]. Immediately after the NLCS-baseline measurement, the subcohort (2411 men, 2589 women) was randomly sampled from the cohort, and actively followed up since 1986 for vital status and migration. For this analysis the final follow-up date was December 31, 1996. Participants who emigrated where censored at migration date. Data on mortality and causes of death in the cohort-at-large were obtained from linkage with the Dutch Central Bureau of Genealogy and Statistics Netherlands. Through this linkage, 18,091 deaths were identified between January 1987 and December 1996. The completeness of the mortality follow-up was 99% [22]. Overall mortality follow-up was not available for the NLCS after this period. Causes of death were coded according to the International Classification of Disease, ninth revision (ICD-9) for 1987–1995 and ICD-10 for 1996 [13]. Besides total mortality, the following major primary causes of death were separately investigated: cancer (ICD-9: 140–239; ICD-10: C00–D48), cardiovascular (CVD) (ICD-9: 390–459; ICD-10: I00–I99), respiratory disease (ICD-9: 460–519; ICD-10: J00–J99).

### Exposure assessment

The baseline questionnaire measured dietary intake (150 items), detailed smoking habits and many other lifestyle factors, and medical conditions [20]. Habitual consumption of food and beverages during the year preceding baseline was assessed using a semi-quantitative food-frequency questionnaire (FFQ), which was validated against a 9-day diet record [23]. Average daily intakes of meat, fish, eggs, dairy, pulses and nuts were calculated by multiplying the intake frequency of individual items by their weights, using either standard serving sizes or reported portion sizes, and summing over the items within these food groups. Fresh (unprocessed) red meat consisted of the following items: beef, pork, minced meat (including beef and pork), liver, and other meat (e.g., horsemeat, lamb). Poultry included chicken and turkey. Processed meat was defined as meat items that had undergone some form of preservation (mostly treatment with nitrite salt, sometimes smoked or fermented). Processed meat consisted of the following items: ham, bacon, smoked beef or pork loin roll, and other sliced cold meats (e.g., sausages). As in the European Prospective Investigation into Cancer (EPIC) [2], processed meat mainly referred to processed red meat, but may contain small amounts of processed white meat (poultry) also, as in sausages for example.

There were 3 items on fish consumption (with the hot meal, for lunch, or as a snack in between meals). Low-fat dairy items included nonfermented and fermented low-fat milk and low-fat cheese [24].

The FFQ has been validated and tested for reproducibility in the NLCS [23, 25]. The Spearman correlation coefficients for fresh meat, processed meat, and fish as assessed by the questionnaire and those estimated from the 9-day record were 0.46, 0.54 and 0.53, respectively. For dairy and eggs, these were 0.60 and 0.61, respectively [23]. No validation data are available for pulses and nut intake [13].

For fresh meat and processed meat together, respondents could also indicate whether they ate “more, less, or the same amount” 5 years before baseline, compared to baseline. Stable meat consumers were those indicating the same amount. Nutrient intakes were calculated using the computerized Dutch food composition table [26].

### Population for analysis

From the 18,091 deaths in the cohort, subjects who reported a history of cancer (excluding skin cancer) or CVD (myocardial infarction, angina pectoris, stroke) at baseline were excluded from this mortality analysis to avoid reverse causation, leaving 12,386 deaths. A similar exclusion applied to the subcohort yielded 4193 subcohort members available. Additionally, subjects with incomplete or inconsistent dietary data were excluded, according to criteria described previously [22, 23], leaving 10,382 deaths (6701 men, 3681 women) and 3693 subcohort members (1743 men, 1950 women) available for analysis after these exclusions. Multivariable case-cohort analyses were based on 8823 deaths and 3202 subcohort members with complete data on diet and confounders. Cause-specific numbers are presented in Supplementary Figure S1.

### Statistical analysis

For the intakes of red meat and processed meat, the mean (SD) values were calculated in the subcohort. Associations between red and processed meat intake and various (non)dietary characteristics were examined by cross-tabulations. The relationship between intake of red meat, processed meat and other protein sources and overall mortality and cause-specific mortality was evaluated using Cox proportional hazards models; deaths due to other causes were censored at date of death for cause-specific analyses. Analyses were initially done for men and women separately to allow and evaluate possible effect modification by sex, but later combined because there was no significant interaction. The proportional hazards assumption was evaluated using –ln(–ln) survival plots, and by adding interaction terms between exposure and time to the multivariable adjusted models, and tested using Wald tests. No violation of the proportional hazards assumption was found. Standard errors were estimated using the robust Huber–White sandwich estimator to account for additional variance introduced by the subcohort sampling [27].

In age–sex and multivariable-adjusted survival analyses, meat and protein source intake were evaluated and tested on categorical (quintiles for red and processed meat; quartiles for low-fat dairy; categories 0, 0.1 to < 10, 10 to < 20, 20 + g/day for poultry, eggs, fish, pulses and nuts) and continuous scales. In multivariable analyses, hazard ratios (HRs) were corrected for potential confounders: age at baseline (continuous, years), cigarette smoking status (coded as never, former, current smoker), number of cigarettes smoked per day, and years of smoking (both continuous, centered), history of physician-diagnosed hypertension (no, yes) and diabetes (no, yes), body height (continuous, m), BMI (< 18.5, 18.5 to < 25, 25 to < 30, ≥ 30 kg/m^2^), non-occupational physical activity (< 30, 30–60, 61–90, ≥ 90 min/day), highest level of education (primary school or lower vocational, secondary or medium vocational, and higher vocational or university), intake of alcohol (0, 0.1 to < 5, 5 to < 15, 15 to < 30, 30 + g/day), vegetables and fruit (both continuous, g/day), nuts (0, 0.1 to < 5, 5 to < 10, 10 + g/day), energy (continuous, kcal/day), use of nutritional supplements (no, yes), and, in women, postmenopausal hormone replacement therapy (never, ever). Red meat and processed meat intake were mutually adjusted for each other as well in additional analyses. Analyses were repeated after excluding deaths occurring in the first 2 years of follow-up. Listwise deletion was applied to handle missing data for potential confounders. Tests for trends were assessed using Wald tests, by fitting median values of intake per intake category as continuous terms. Tests for non-linearity in the associations with mortality were conducted using restricted cubic splines, using three knots (10th, 50th, 90th percentiles). These survival analyses were carried out for overall mortality, followed by cause-specific analyses.

Substitution analyses were performed in which mortality associations were estimated when replacing processed meat with other dietary protein sources. The multivariable model (with the covariables mentioned earlier) included intake of all groups of dietary protein sources as continuous variables (units 50 g/day), except for processed meat which is to be replaced, and a variable representing the total intake of dietary protein sources (unit 50 g/day). Then, hazard ratios for each of the dietary protein sources in the model can be interpreted as the estimated difference in rate of mortality associated with 50 g/day higher intake of the protein sources included in the model and a concomitant lower intake of the source left out of the model, i.e. processed meat [28, 29], so that the effect of substituting equal amounts, e.g., replacing 50 g/day of processed meat with 50 g/d of poultry, can be estimated.

To evaluate potential residual confounding by mortality risk factors, and interactions, analyses for overall mortality were also conducted in subgroups of smoking, alcohol, BMI, and physical activity. Multiplicative interactions with these factors were tested using Wald tests and cross-product terms. In sensitivity analyses, we additionally adjusted for heme iron intake [30] and nitrite intake [31] to investigate whether associations with red meat and processed meat, respectively, might be attributed to these compounds. The heme iron content from meat items and the meat used in mixed dishes was estimated as an animal-specific percentage of total iron, derived from data in the literature, and has been reported in detail elsewhere [30]. Food composition values for nitrite were obtained from analyses conducted by the Dutch National Public Health Institute in 1984, based on 5 samples per product [31]. All analyses were performed using Stata version 14; presented *P* values are two-sided.

## Results

The mean (SD) intake of unprocessed red meat among subcohort members was 94.0 (41.5) g/day in men and 88.8 (37.7) g/day in women; for processed meat, these values were 16.3 (17.3) and 10.5 (11.6) g/day, respectively. Among subcohort members, both higher red meat and processed meat consumption were associated with higher alcohol intake and BMI in men and women, and red meat was associated with lower fruit and low-fat dairy intake (Table 1). Among men consuming more red meat and processed meat, there were fewer never smokers, and fewer people had university or higher vocational education. In men, red meat was further positively associated with intake of vegetables, eggs and nuts; in women, red meat was positively associated with diabetes, and inversely with poultry intake, while processed meat was positively associated with poultry. The Spearman correlation coefficients between unprocessed red meat and processed meat were rather low: 0.17 in men and 0.18 in women.

**Table 1:** Baseline characteristics (means, or percent) according to red meat and processed meat intake in male and female subcohort members with complete dietary and covariable data, Netherlands Cohort Study

Characteristic	Red meat (quintiles)					Processed meat (quintiles)				
	1	2	3	4	5	1	2	3	4	5
*Men*										
Median (g/day)	46.4	73.2	89.4	108.8	144.8	0.0	5.5	11.5	19.2	36.0
N	304	311	303	307	310	294	312	308	320	301
Age, mean (year)	61.7	61.5	61.3	61.3	60.7	62.3	61.3	61.0	61.1	60.8
BMI (kg/m^2^)	24.4	24.8	24.7	25.2	25.3	24.5	24.9	24.8	25.0	25.1
Physical activity, nonoccupational (min/day)	81.8	78.6	80.8	83.9	78.5	81.8	79.2	74.0	82.4	86.3
Alcohol intake (g/day)	11.9	14.7	14.7	15.7	18.6	12.7	13.9	16.6	16.3	16.1
Vegetable intake (g/day)	180.3	175.5	181.8	185.6	209.3	187.1	191.7	176.5	190.4	186.7
Fruit intake (g/day)	175.1	155.6	150.2	151.1	147.8	165.4	151.6	145.2	155.7	162.3
Poultry intake (g/day)	13.9	14.9	13.3	11.1	13.1	13.1	12.6	13.7	12.8	14.1
Eggs intake (g/day)	15.7	16.5	16.9	17.5	19.0	14.8	16.1	18.0	17.7	19.1
Fish intake (g/day)	16.3	12.7	13.2	13.3	13.8	13.0	13.9	14.1	14.0	14.3
Pulses intake (g/day)	9.5	9.8	9.5	8.4	10.3	10.5	10.0	8.4	8.4	10.3
Nut intake (g/day)	7.3	7.6	7.3	9.8	9.4	5.7	8.6	9.6	8.6	8.8
Low-fat dairy (g/day)	217.1	184.6	176.2	162.8	149.0	183.8	187.5	172.8	172.2	173.0
Never smoker (%)	16.1	14.5	14.9	13.4	13.5	18.4	14.1	13.0	14.1	13.0
University or higher vocational education (%)	24.3	22.8	21.5	20.8	17.4	23.1	23.7	23.7	18.8	17.6
Diabetes (%)	2.0	2.9	4.3	3.3	2.3	3.4	1.0	1.6	3.4	5.3
Hypertension (%)	23.7	24.1	21.5	21.5	21.3	24.5	23.7	23.4	21.3	19.3
Nutritional supplement user (%)	28.9	23.8	20.5	20.8	23.2	27.9	20.2	23.7	26.6	18.9
*Women*										
Median (g/day)	37.3	61.9	79.2	95.7	128.6	0.0	2.6	6.8	12.8	25.3
N	336	334	338	337	322	342	294	352	341	338
Age, mean (year)	61.7	61.5	61.7	61.5	61.4	61.9	61.9	61.2	61.5	61.3
BMI (kg/m^2^)	24.2	24.7	25.1	25.2	25.8	24.5	24.7	25.3	25.3	25.2
Physical activity, nonoccupational (min/day)	66.4	63.3	69.0	63.8	68.9	64.8	65.1	68.3	61.5	71.5
Alcohol intake (g/day)	4.6	6.0	5.9	6.7	7.5	4.9	6.4	6.2	6.9	6.4
Vegetable intake (g/day)	192.6	190.3	188.2	194.0	200.2	190.9	193.3	194.4	190.3	196.1
Fruit intake (g/day)	198.3	200.6	201.1	188.8	188.6	195.8	199.1	193.2	188.0	202.3
Poultry intake (g/day)	14.7	15.6	12.8	10.5	11.9	11.0	11.3	14.3	14.6	14.1
Eggs intake (g/day)	12.7	13.7	15.5	14.9	17.0	13.0	14.3	14.5	16.5	15.5
Fish intake (g/day)	11.8	11.6	11.2	10.4	11.3	9.7	10.8	11.6	11.9	12.2
Pulses intake (g/day)	8.7	6.6	6.2	6.2	6.1	7.8	7.0	6.2	7.1	5.8
Nut intake (g/day)	4.4	3.8	4.0	5.0	5.4	3.8	4.9	4.9	4.9	4.1
Low-fat dairy (g/day)	221.6	195.6	196.0	211.4	178.7	209.0	204.7	206.5	187.3	196.9
Never smoker (%)	62.2	56.9	59.5	57.9	58.4	62.3	58.5	54.0	59.5	60.7
University or higher vocational education (%)	12.2	11.7	8.0	12.2	7.1	10.8	11.9	7.7	10.0	11.2
Diabetes (%)	1.8	1.8	3.3	3.6	4.7	2.6	2.0	2.0	2.1	6.2
Hypertension (%)	24.4	26.6	32.2	25.8	26.7	26.6	28.9	26.1	28.2	26.3
Nutritional supplement user (%)	51.8	40.1	30.2	34.7	31.7	41.8	36.7	40.1	37.8	32.0
Ever used hormone replacement therapy (%)	13.7	13.2	13.6	14.5	11.5	15.5	12.9	11.6	12.9	13.6

Of the 8823 deaths with complete information on dietary intake and potential confounders, 5797 occurred in men and 3026 in women. Information on baseline exposure and covariable characteristics of deceased subjects versus subcohort members is presented in Supplementary Table 1. Multivariable-adjusted survival analyses showed no significant heterogeneity between men and women; therefore results are presented for men and women combined. Red meat (unprocessed) intake was not significantly associated with overall mortality, nor with cause-specific mortality (Table 2), in both categorical and continuous multivariable-adjusted analyses. Among the 8823 total deaths with complete information, there were 3917 deaths due to cancer, 2985 cardiovascular deaths and 550 deaths due to respiratory disease. The positive association with cancer mortality in age–sex-adjusted analyses disappeared after multivariable adjustment. Processed meat intake showed a statistically significantly positive association with overall mortality in multivariable-adjusted analyses (Table 2), with a HR (95% CI) of 1.21 (1.02–1.44) when comparing the highest versus lowest intake quintile (P_trend_ = 0.049). These associations were essentially similar when analyses were limited to stable meat users in sensitivity analyses (Table 2).

**Table 2:** Overall and cause-specific mortality according to red and processed meat intake in multivariable-adjusted analyses, NLCS

	Quintile of intake						*P* trend	Continuous, per 50 g/d
	Q1 (ref)	Q2	Q3	Q4	Q5			
**Red meat** (**nonprocessed**)								
Median intake (g/day)	41.3	66.9	84.3	101.4	140.4			
Person-years in subcohort	6077	6014	6220	6034	5975			30,320
*Total mortality*								
No. of deaths	1622	1708	1678	1971	1844			8823
Age–sex-adjusted HR (95% CI)	1	1.04 (0.90–1.21)	0.96 (0.83–1.11)	1.10 (0.95–1.28)	1.06 (0.92–1.23)		0.323	1.04 (0.98–1.10)
Multivariable-adjusted HR (95% CI)	1	1.02 (0.87–1.21)	0.92 (0.79–1.09)	1.07 (0.91–1.26)	1.02 (0.86–1.20)		0.709	1.03 (0.97–1.10)
Stable meat users, no. of deaths	955	1213	1296	1563	1492			6519
Multivariable-adjusted HR (95% CI)	1	0.93 (0.76–1.15)	0.86 (0.70–1.05)	1.01 (0.83–1.23)	0.92 (0.75–1.12)		0.710	1.01 (0.93–1.09)
*Cancer mortality*								
No. of deaths	693	710	757	930	827			3917
Age–sex-adjusted HR (95% CI)	1	1.02 (0.86–1.20)	1.02 (0.87–1.19)	1.23 (1.05–1.44)	1.12 (0.95–1.31)		0.049	1.07 (1.01–1.14)
Multivariable-adjusted HR (95% CI)	1	1.00 (0.84–1.20)	0.97 (0.82–1.15)	1.17 (0.99–1.39)	1.02 (0.85–1.21)		0.518	1.04 (0.97–1.11)
*Cardiovascular disease mortality*								
No. of deaths	562	610	574	630	609			2985
Age–sex-adjusted HR (95% CI)	1	1.07 (0.89–1.28)	0.94 (0.79–1.12)	1.00 (0.84–1.20)	1.00 (0.84–1.20)		0.820	1.01 (0.94–1.09)
Multivariable-adjusted HR (95% CI)	1	1.02 (0.83–1.25)	0.88 (0.72–1.08)	0.96 (0.79–1.18)	0.95 (0.77–1.17)		0.584	1.00 (0.92–1.08)
*Respiratory mortality*								
No. of deaths	109	107	98	123	113			550
Age–sex-adjusted HR (95% CI)	1	0.96 (0.70–1.31)	0.81 (0.59–1.11)	0.97 (0.72–1.31)	0.93 (0.69–1.27)		0.759	1.01 (0.89–1.14)
Multivariable-adjusted HR (95% CI)	1	1.20 (0.81–1.78)	1.04 (0.71–1.53)	1.20 (0.84–1.72)	1.23 (0.84–1.80)		0.318	1.13 (0.97–1.31)
**Processed meat**								
Median intake (g/day)	0	4.3	9.1	16.0	30.8			
Person-years in subcohort	5918	6171	5977	6095	6159			30,320
*Total mortality*								
No. of deaths	1581	1724	1589	1871	2058			8823
Age–sex-adjusted HR (95% CI)	1	1.09 (0.94–1.27)	1.01 (0.87–1.17)	1.16 (1.00–1.34)	1.18 (1.02–1.37)		0.022	1.11 (0.95–1.29)
Multivariable-adjusted HR (95% CI)	1	1.16 (0.99–1.37)	1.06 (0.89–1.25)	1.24 (1.05–1.46)	1.21 (1.02–1.44)		0.049	1.12 (0.94–1.34)
Stable meat users, no. of deaths	1035	1200	1178	1434	1672			6519
Multivariable-adjusted HR (95% CI)	1	1.12 (0.92–1.37)	1.00 (0.81–1.22)	1.21 (0.99–1.47)	1.22 (0.99–1.50)		0.045	1.14 (0.94–1.39)
*Cancer mortality*								
No. of deaths	699	807	692	796	923			3917
Age–sex-adjusted HR (95% CI)	1	1.14 (0.97–1.34)	0.98 (0.83–1.16)	1.10 (0.93–1.29)	1.18 (1.01–1.39)		0.065	1.03 (0.88–1.22)
Multivariable-adjusted HR (95% CI)	1	1.14 (0.96–1.35)	0.97 (0.81–1.16)	1.10 (0.92–1.30)	1.16 (0.97–1.39)		0.154	1.00 (0.83–1.20)
*Cardiovascular disease mortality*								
No. of deaths	525	554	559	661	686			2985
Age–sex-adjusted HR (95% CI)	1	1.07 (0.89–1.29)	1.08 (0.90–1.30)	1.24 (1.03–1.49)	1.19 (0.99–1.43)		0.039	1.17 (0.98–1.40)
Multivariable-adjusted HR (95% CI)	1	1.16 (0.95–1.43)	1.15 (0.93–1.42)	1.38 (1.12–1.71)	1.26 (1.01–1.56)		0.047	1.24 (1.01–1.53)
Respiratory mortality								
No. of deaths	91	94	97	126	142			550
Age–sex-adjusted HR (95% CI)	1	1.07 (0.77–1.48)	1.10 (0.79–1.53)	1.37 (1.00–1.89)	1.42 (1.04–1.93)		0.009	1.26 (0.98–1.63)
Multivariable-adjusted HR (95% CI)	1	1.44 (0.98–2.13)	1.39 (0.94–2.06)	1.80 (1.24–2.62)	1.79 (1.19–2.67)		0.007	1.58 (1.14–2.19)

Multivariable analyses were adjusted for: age at baseline (continuous, in years), sex, cigarette smoking status (coded as never, former, current smoker), number of cigarettes smoked per day, and years of smoking (both continuous, centered), history of physician-diagnosed hypertension (no, yes) and diabetes (no, yes), body height (continuous, m), BMI (< 18.5, 18.5 to < 25, 25 to < 30, ≥ 30 kg/m^2^), non-occupational physical activity (< 30, 30–60, 61–90, ≥ 90 min/day), highest level of education (primary school or lower vocational, secondary or medium vocational, and higher vocational or university), intake of alcohol (0, 0.1 to < 5, 5 to < 15, 15 to < 30, 30 + g/day), vegetables and fruit (both continuous, g/day), energy (continuous, kcal/day), use of nutritional supplements (no, yes), and, in women, postmenopausal HRT (never, ever)

**Fig. 1:**
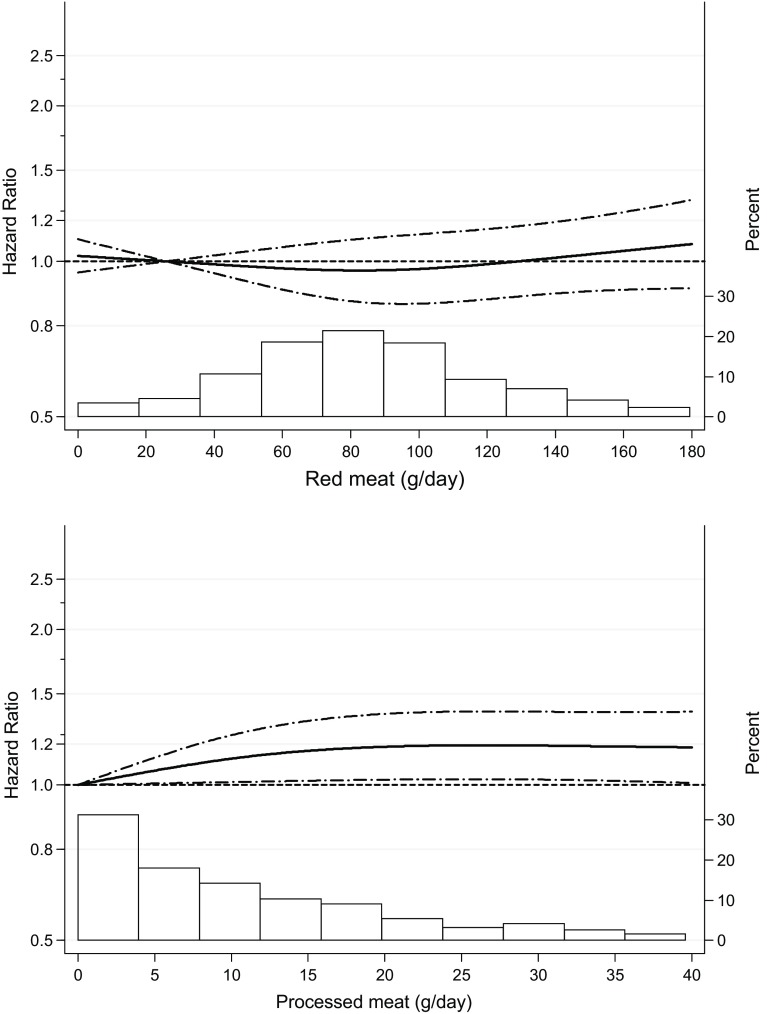
Spline regression curves for the association between (unprocessed) red meat (upper panel) and processed meat (lower panel) intake and total mortality. Solid lines represents point estimates and dashed lines represent 95% confidence intervals. Multivariable HRs were calculated by restricted cubic spline regression (using 3 knots at 10th, 50th, and 90th percentiles) adjusting for: age at baseline (continuous, in years), cigarette smoking status (coded as never, former, current smoker), number of cigarettes smoked per day, and years of smoking (both continuous, centered), history of physician-diagnosed hypertension (no, yes) and diabetes (no, yes), body height (continuous, m), BMI (< 18.5, 18.5 to < 25, 25 to < 30, ≥ 30 kg/m^2^), non-occupational physical activity (< 30, 30–60, 61–90, ≥ 90 min/day), highest level of education (primary school or lower vocational, secondary or medium vocational, and higher vocational or university), intake of alcohol (0, 0.1 to < 5, 5 to < 15, 15 to < 30, 30 + g/day), vegetables and fruit (both continuous, g/day), energy (continuous, kcal/day), use of nutritional supplements (no, yes), and, in women, postmenopausal HRT (never, ever). The histograms show the percentage of participants (right *y* axis) consuming each level of red meat and processed meat, respectively

**Fig. 2:**
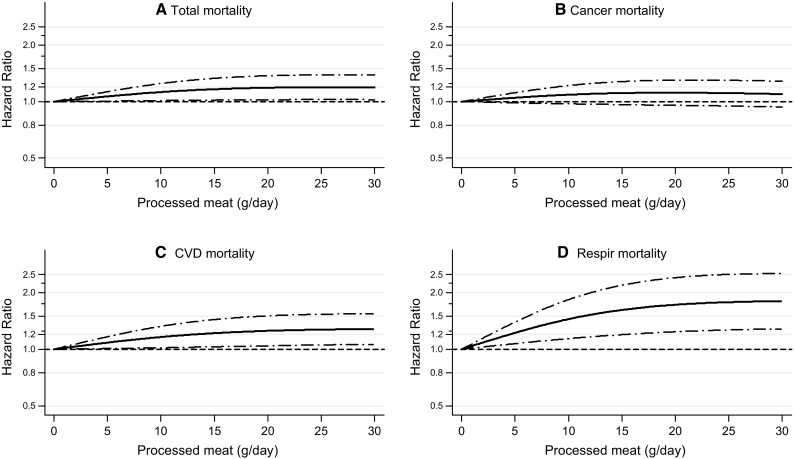
Spline regression curves for the association between processed meat intake and **a** total mortality, **b** cancer mortality, **c** CVD mortality, **d** respiratory mortality. Multivariable HRs were calculated by restricted cubic spline regression (using 3 knots at 10th, 50th, and 90th percentiles) adjusting for: age at baseline (continuous, in years), cigarette smoking status (coded as never, former, current smoker), number of cigarettes smoked per day, and years of smoking (both continuous, centered), history of physician-diagnosed hypertension (no, yes) and diabetes (no, yes), body height (continuous, m), BMI (< 18.5, 18.5 to < 25, 25 to < 30, ≥ 30 kg/m^2^), non-occupational physical activity (< 30, 30–60, 61–90, ≥ 90 min/day), highest level of education (primary school or lower vocational, secondary or medium vocational, and higher vocational or university), intake of alcohol (0, 0.1 to < 5, 5 to < 15, 15 to < 30, 30 + g/day), vegetables and fruit (both continuous, g/day), energy (continuous, kcal/day), use of nutritional supplements (no, yes), and, in women, postmenopausal HRT (never, ever)

Regarding cause-specific mortality, processed meat intake was also significantly associated with CVD mortality, with a HR (95% CI) of 1.26 (1.01–1.56) when comparing highest versus lowest quintile (P_trend_ = 0.047), and with respiratory mortality, with HR (95% CI) of 1.79 (1.19–2.67) and P_trend_ = 0.007, but not significantly with cancer mortality. Significant associations were also observed with CVD and respiratory mortality when processed meat was modeled as continuous exposure (Table 2). Results were similar after mutual adjustment, i.e. when red meat analyses were additionally adjusted for processed meat intake, and vice versa (data not shown). Spline regression plots of total mortality in relation to red meat and processed meat are shown in Fig. 1. There was no statistical evidence for nonlinearity for red and processed meat (*P* for nonlinearity = 0.279 and 0.064, respectively). Spline regression of cause-specific mortality (Fig. 2) revealed only evidence for nonlinearity for processed meat and respiratory deaths (*P* for nonlinearity = 0.016).

Results of Cox regression analyses for other dietary protein sources are shown in Tables 3 and 4. Intake values of poultry (unprocessed), eggs, fish, pulses, and nuts were categorized into 0, 0.1 to < 10, 10.0 to < 20, and 20 + g/day (for reasons of comparability). For low-fat dairy, quartiles of intake were used. Poultry intake was significantly inversely related to cancer mortality in multivariable-adjusted analyses (P_trend_ = 0.006), and less clearly with overall mortality (P_trend_ = 0.044) (Table 3). Subjects consuming < 20 g/d of eggs showed significantly inverse associations with overall and cancer mortality compared to nonconsumers, but the trend tests or continuous analyses showed no significant associations. No associations with poultry or egg intake were seen for CVD or respiratory mortality. Fish intake was significantly positively associated with overall and cause-specific mortality with significantly elevated HRs for those eating 20 + versus 0 g fish/day ranging from 1.29 to 1.50; only for cancer mortality the trend test or continuous analyses showed no significance.

**Table 3:** Overall and cause-specific mortality according to intake of poultry, eggs and fish in multivariable-adjusted analyses, NLCS

	Category of intake (g/day)				*P* trend	Continuous, per 50 g/d
	0 (ref)	< 10	< 20	20 +		
**Poultry**						
Median intake (g/day)	0	4.3	13.2	22.8		
Person-years in subcohort	6940	7333	7433	8614		30,320
*Total mortality*						
No. of deaths	2222	2259	2027	2315		8823
Age–sex-adjusted HR (95% CI)	1	1.02 (0.89–1.16)	0.90 (0.79–1.03)	0.88 (0.77–1.00)	0.013	0.92 (0.78–1.08)
Multivariable-adjusted HR (95% CI)	1	1.01 (0.87–1.16)	0.90 (0.77–1.04)	0.89 (0.77–1.03)	0.044	0.92 (0.76–1.10)
*Cancer mortality*						
No. of deaths	987	1032	888	1010		3917
Age–sex-adjusted HR (95% CI)	1	1.04 (0.90–1.20)	0.88 (0.76–1.01)	0.86 (0.74–0.98)	0.004	0.82 (0.68–0.99)
Multivariable-adjusted HR (95% CI)	1	1.03 (0.88–1.19)	0.86 (0.74–1.01)	0.85 (0.73–0.99)	0.006	0.81 (0.66–0.99)
*CVD mortality*						
No. of deaths	746	740	690	809		2985
Age–sex-adjusted HR (95% CI)	1	1.00 (0.85–1.18)	0.92 (0.78–1.09)	0.92 (0.79–1.08)	0.215	1.01 (0.83–1.23)
Multivariable-adjusted HR (95% CI)	1	0.94 (0.78–1.12)	0.87 (0.73–1.05)	0.89 (0.75–1.06)	0.183	0.97 (0.77–1.22)
*Respiratory mortality*						
No. of deaths	143	134	138	135		550
Age–sex-adjusted HR (95% CI)	1	0.96 (0.73–1.26)	0.98 (0.74–1.28)	0.82 (0.62–1.07)	0.152	0.96 (0.65–1.42)
Multivariable-adjusted HR (95% CI)	1	1.01 (0.73–1.39)	1.16 (0.83–1.60)	0.95 (0.68–1.32)	0.884	0.94 (0.58–1.52)
**Eggs**						
Median intake (g/day)	0	7.1	14.2	21.4		
Person-years in subcohort	2275	7350	10,602	10,093		30,320
*Total mortality*						
No. of deaths	748	1912	2988	3175		8823
Age–sex-adjusted HR (95% CI)	1	0.74 (0.61–0.89)	0.81 (0.68–0.97)	0.87 (0.73–1.05)	0.429	1.07 (0.88–1.31)
Multivariable-adjusted HR (95% CI)	1	0.78 (0.64–0.96)	0.83 (0.68–1.01)	0.87 (0.71–1.06)	0.990	0.94 (0.75–1.17)
*Cancer mortality*						
No. of deaths	344	820	1314	1439		3917
Age–sex-adjusted HR (95% CI)	1	0.70 (0.57–0.85)	0.78 (0.64–0.95)	0.87 (0.72–1.06)	0.265	1.13 (0.91–1.40)
Multivariable-adjusted HR (95% CI)	1	0.72 (0.58–0.89)	0.78 (0.63–0.96)	0.82 (0.67–1.02)	0.945	0.92 (0.73–1.17)
*CVD mortality*						
No. of deaths	232	670	1014	1069		2985
Age–sex-adjusted HR (95% CI)	1	0.82 (0.65–1.03)	0.88 (0.70–1.10)	0.93 (0.75–1.17)	0.464	1.05 (0.82–1.33)
Multivariable-adjusted HR (95% CI)	1	0.89 (0.69–1.16)	0.90 (0.70–1.16)	0.92 (0.71–1.19)	0.876	0.92 (0.70–1.20)
*Respiratory mortality*						
No. of deaths	49	127	182	192		550
Age–sex-adjusted HR (95% CI)	1	0.71 (0.48–1.05)	0.73 (0.50–1.06)	0.77 (0.53–1.11)	0.614	0.81 (0.55–1.20)
Multivariable-adjusted HR (95% CI)	1	0.97 (0.61–1.53)	0.91 (0.58–1.44)	1.01 (0.64–1.59)	0.892	0.85 (0.55–1.33)
**Fish**						
Median intake (g/day)	0	4.6	14.8	29.8		
Person-years in subcohort	8701	6694	9372	5553		30,320
*Total mortality*						
No. of deaths	2231	2125	2564	1903		8823
Age–sex-adjusted HR (95% CI)	1	1.22 (1.07–1.38)	1.06 (0.94–1.19)	1.28 (1.11–1.46)	0.012	1.21 (1.04–1.40)
Multivariable-adjusted HR (95% CI)	1	1.26 (1.09–1.46)	1.11 (0.97–1.27)	1.34 (1.15–1.56)	0.003	1.22 (1.04–1.44)
*Cancer mortality*						
No. of deaths	962	968	1145	842		3917
Age–sex-adjusted HR (95% CI)	1	1.29 (1.12–1.49)	1.09 (0.96–1.24)	1.31 (1.13–1.52)	0.015	1.18 (1.01–1.40)
Multivariable-adjusted HR (95% CI)	1	1.33 (1.14–1.55)	1.11 (0.96–1.28)	1.29 (1.09–1.51)	0.066	1.13 (0.95–1.35)
*CVD mortality*						
No. of deaths	733	723	861	668		2985
Age–sex-adjusted HR (95% CI)	1	1.25 (1.06–1.46)	1.08 (0.93–1.25)	1.35 (1.15–1.60)	0.007	1.28 (1.07–1.54)
Multivariable-adjusted HR (95% CI)	1	1.27 (1.06–1.52)	1.16 (0.98–1.37)	1.45 (1.20–1.74)	0.001	1.30 (1.07–1.58)
*Respiratory mortality*						
No. of deaths	149	144	138	119		550
Age–sex-adjusted HR (95% CI)	1	1.20 (0.92–1.57)	0.85 (0.65–1.10)	1.17 (0.88–1.54)	0.823	1.18 (0.85–1.64)
Multivariable-adjusted HR (95% CI)	1	1.41 (1.02–1.94)	1.03 (0.75–1.42)	1.50 (1.08–2.10)	0.113	1.43 (1.00–2.03)

Multivariable analyses were adjusted for: age at baseline (continuous, in years), sex, cigarette smoking status (coded as never, former, current smoker), number of cigarettes smoked per day, and years of smoking (both continuous, centered), history of physician-diagnosed hypertension (no, yes) and diabetes (no, yes), body height (continuous, m), BMI (< 18.5, 18.5 to < 25, 25 to < 30, ≥ 30 kg/m^2^), non-occupational physical activity (< 30, 30–60, 61–90, ≥ 90 min/day), highest level of education (primary school or lower vocational, secondary or medium vocational, and higher vocational or university), intake of alcohol (0, 0.1 to < 5, 5 to < 15, 15 to < 30, 30 + g/day), vegetables and fruit (both continuous, g/day), energy (continuous, kcal/day), use of nutritional supplements (no, yes), and, in women, postmenopausal HRT (never, ever)

**Table 4:** Overall and cause-specific mortality according to intake of nuts, pulses and low-fat dairy in multivariable-adjusted^a^ analyses, NLCS

	Category of intake (g/day)				P trend	Continuous, per 50 g/d
	0 (ref)	< 10	< 20	20 +		
**Pulses**						
Median intake (g/day)	0	4.3	16.0	27.8		
Person-years in subcohort	11,633	9042	6500	3145		30,320
*Total mortality*						
No. of deaths	3371	2546	1914	992		8823
Age–sex-adjusted HR (95% CI)	1	0.97 (0.87–1.08)	1.03 (0.91–1.17)	0.98 (0.83–1.14)	0.863	1.00 (0.82–1.22)
Multivariable-adjusted HR (95% CI)	1	1.03 (0.91–1.17)	1.11 (0.97–1.27)	1.01 (0.85–1.21)	0.469	1.04 (0.83–1.30)
*Cancer mortality*						
No. of deaths	1439	1168	864	446		3917
Age–sex-adjusted HR (95% CI)	1	1.04 (0.92–1.17)	1.09 (0.95–1.25)	1.04 (0.87–1.23)	0.427	1.09 (0.88–1.36)
Multivariable-adjusted HR (95% CI)	1	1.09 (0.95–1.24)	1.12 (0.97–1.29)	1.03 (0.85–1.25)	0.508	1.07 (0.84–1.37)
*CVD mortality*						
No. of deaths	1183	804	661	337		2985
Age–sex-adjusted HR (95% CI)	1	0.87 (0.76–1.00)	1.02 (0.87–1.18)	0.93 (0.77–1.12)	0.984	0.96 (0.75–1.23)
Multivariable-adjusted HR (95% CI)	1	0.91 (0.78–1.07)	1.09 (0.93–1.29)	0.99 (0.80–1.23)	0.449	1.01 (0.76–1.33)
*Respiratory mortality*						
No. of deaths	206	170	120	54		550
Age–sex-adjusted HR (95% CI)	1	1.04 (0.83–1.32)	1.05 (0.81–1.36)	0.83 (0.59–1.16)	0.410	0.75 (0.50–1.13)
Multivariable-adjusted HR (95% CI)	1	1.26 (0.95–1.66)	1.28 (0.94–1.75)	0.93 (0.62–1.38)	0.927	0.89 (0.57–1.40)
**Nuts**						
Median intake (g/day)	0	3.3	12.8	28.5		
Person-years in subcohort	10,518	14,260	2862	2680		30,320
*Total mortality*						
No. of deaths	3732	3696	684	711		8823
Age–sex-adjusted HR (95% CI)	1	0.77 (0.69–0.85)	0.69 (0.59–0.82)	0.70 (0.59–0.83)	< 0.001	0.63 (0.51–0.79)
Multivariable-adjusted HR (95% CI)	1	0.84 (0.75–0.95)	0.77 (0.64–0.93)	0.78 (0.64–0.95)	0.010	0.70 (0.56–0.89)
*Cancer mortality*						
No. of deaths	1556	1710	308	343		3917
Age–sex-adjusted HR (95% CI)	1	0.84 (0.75–0.94)	0.74 (0.61–0.89)	0.80 (0.66–0.97)	0.013	0.72 (0.57–0.91)
Multivariable-adjusted HR (95% CI)	1	0.90 (0.80–1.01)	0.75 (0.61–0.93)	0.83 (0.68–1.03)	0.044	0.72 (0.56–0.93)
*CVD mortality*						
No. of deaths	1281	1223	236	245		2985
Age–sex-adjusted HR (95% CI)	1	0.75 (0.66–0.85)	0.71 (0.58–0.87)	0.70 (0.57–0.87)	0.002	0.62 (0.47–0.82)
Multivariable-adjusted HR (95% CI)	1	0.85 (0.74–0.98)	0.83 (0.66–1.05)	0.84 (0.66–1.07)	0.202	0.75 (0.57–1.00)
*Respiratory mortality*						
No. of deaths	284	197	42	27		550
Age–sex-adjusted HR (95% CI)	1	0.54 (0.44–0.67)	0.56 (0.39–0.81)	0.33 (0.22–0.52)	< 0.001	0.22 (0.10–0.46)
Multivariable-adjusted HR (95% CI)	1	0.65 (0.51–0.84)	0.73 (0.48–1.13)	0.48 (0.30–0.78)	0.009	0.39 (0.19–0.81)

**Low**-**fat dairy** (*quartiles*)	Q1 (ref)	Q2	Q3	Q4		
Median intake (g/day)	0	85.4	203.3	392.9		
Person-years in subcohort	7449	7595	7583	7693		30,320
*Total mortality*						
No. of deaths	2524	2161	2072	2066		8823
Age–sex-adjusted HR (95% CI)	1	0.86 (0.76–0.98)	0.81 (0.71–0.92)	0.86 (0.76–0.98)	0.034	0.99 (0.97–1.00)
Multivariable-adjusted HR (95% CI)	1	0.95 (0.83–1.09)	0.86 (0.75–0.99)	0.96 (0.83–1.11)	0.435	0.99 (0.98–1.01)
*Cancer mortality*						
No. of deaths	1116	948	931	922		3917
Age–sex-adjusted HR (95% CI)	1	0.86 (0.75–0.99)	0.82 (0.72–0.95)	0.87 (0.75–1.00)	0.071	0.98 (0.97–1.00)
Multivariable-adjusted HR (95% CI)	1	0.94 (0.81–1.09)	0.88 (0.76–1.02)	1.02 (0.87–1.18)	0.896	1.00 (0.98–1.01)
*CVD mortality*						
No. of deaths	833	744	720	688		2985
Age–sex-adjusted HR (95% CI)	1	0.90 (0.77–1.06)	0.85 (0.73–0.99)	0.88 (0.75–1.03)	0.128	0.99 (0.97–1.00)
Multivariable-adjusted HR (95% CI)	1	0.98 (0.82–1.16)	0.88 (0.74–1.05)	0.92 (0.77–1.11)	0.271	0.99 (0.97–1.01)
*Respiratory mortality*						
No. of deaths	181	141	117	111		550
Age–sex-adjusted HR (95% CI)	1	0.78 (0.60–1.02)	0.63 (0.48–0.83)	0.67 (0.51–0.88)	0.003	0.96 (0.93–0.99)
Multivariable-adjusted HR (95% CI)	1	0.97 (0.72–1.30)	0.75 (0.55–1.02)	0.83 (0.60–1.15)	0.143	0.98 (0.95–1.02)

Multivariable analyses were adjusted for: age at baseline (continuous, in years), sex, cigarette smoking status (coded as never, former, current smoker), number of cigarettes smoked per day, and years of smoking (both continuous, centered), history of physician-diagnosed hypertension (no, yes) and diabetes (no, yes), body height (continuous, m), BMI (< 18.5, 18.5 to < 25, 25 to < 30, ≥ 30 kg/m^2^), non-occupational physical activity (< 30, 30–60, 61–90, ≥ 90 min/day), highest level of education (primary school or lower vocational, secondary or medium vocational, and higher vocational or university), intake of alcohol (0, 0.1 to < 5, 5 to < 15, 15 to < 30, 30 + g/day), vegetables and fruit (both continuous, g/day), energy (continuous, kcal/day), use of nutritional supplements (no, yes), and, in women, postmenopausal HRT (never, ever)

While intake of pulses or low-fat dairy seemed unrelated to overall and cause-specific mortality (Table 4), nut intake showed mostly significantly inverse associations with mortality.

**Fig. 3:**
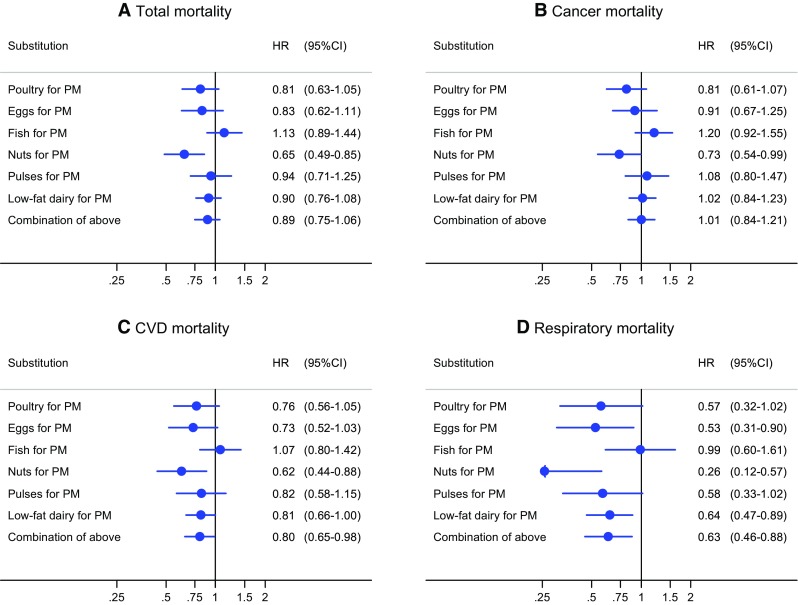
Hazard ratios and 95% CIs (error bars) for **a** total mortality, **b** cancer mortality, **c** CVD mortality, **d** respiratory mortality associated with substituting 50 g/day of processed meat (PM) with 50 g/day of other protein sources. Multivariable HRs were calculated by restricted cubic spline regression (using 3 knots at 10th, 50th, and 90th percentiles) adjusting for: age at baseline (continuous, in years), cigarette smoking status (coded as never, former, current smoker), number of cigarettes smoked per day, and years of smoking (both continuous, centered), history of physician-diagnosed hypertension (no, yes) and diabetes (no, yes), body height (continuous, m), BMI (< 18.5, 18.5 to < 25, 25 to < 30, ≥ 30 kg/m^2^), non-occupational physical activity (< 30, 30–60, 61–90, ≥ 90 min/day), highest level of education (primary school or lower vocational, secondary or medium vocational, and higher vocational or university), intake of alcohol (0, 0.1 to < 5, 5 to < 15, 15 to < 30, 30 + g/day), vegetables and fruit (both continuous, g/day), energy (continuous, kcal/day), use of nutritional supplements (no, yes), and, in women, postmenopausal HRT (never, ever)

In substitution analyses (Fig. 3), replacing 50 g/day of processed meat with 50 g/day of a combination of poultry, eggs, fish, pulses, nuts and low-fat dairy was associated with a 11% lower risk of overall mortality [HR 0.89 (95% CI, 0.75–1.06)], 20% lower risk of cardiovascular mortality [HR 0.80 (95% CI, 0.65–0.98)], and 37% lower risk of respiratory mortality [HR 0.63 (95% CI, 0.46–0.88)]. Nuts appeared to an important contributor to the beneficial substitution effect for all endpoints (Fig. 3), whereas replacement of processed meat with eggs and low-fat dairy contributed also beneficially for respiratory and CVD mortality.

**Fig. 4:**
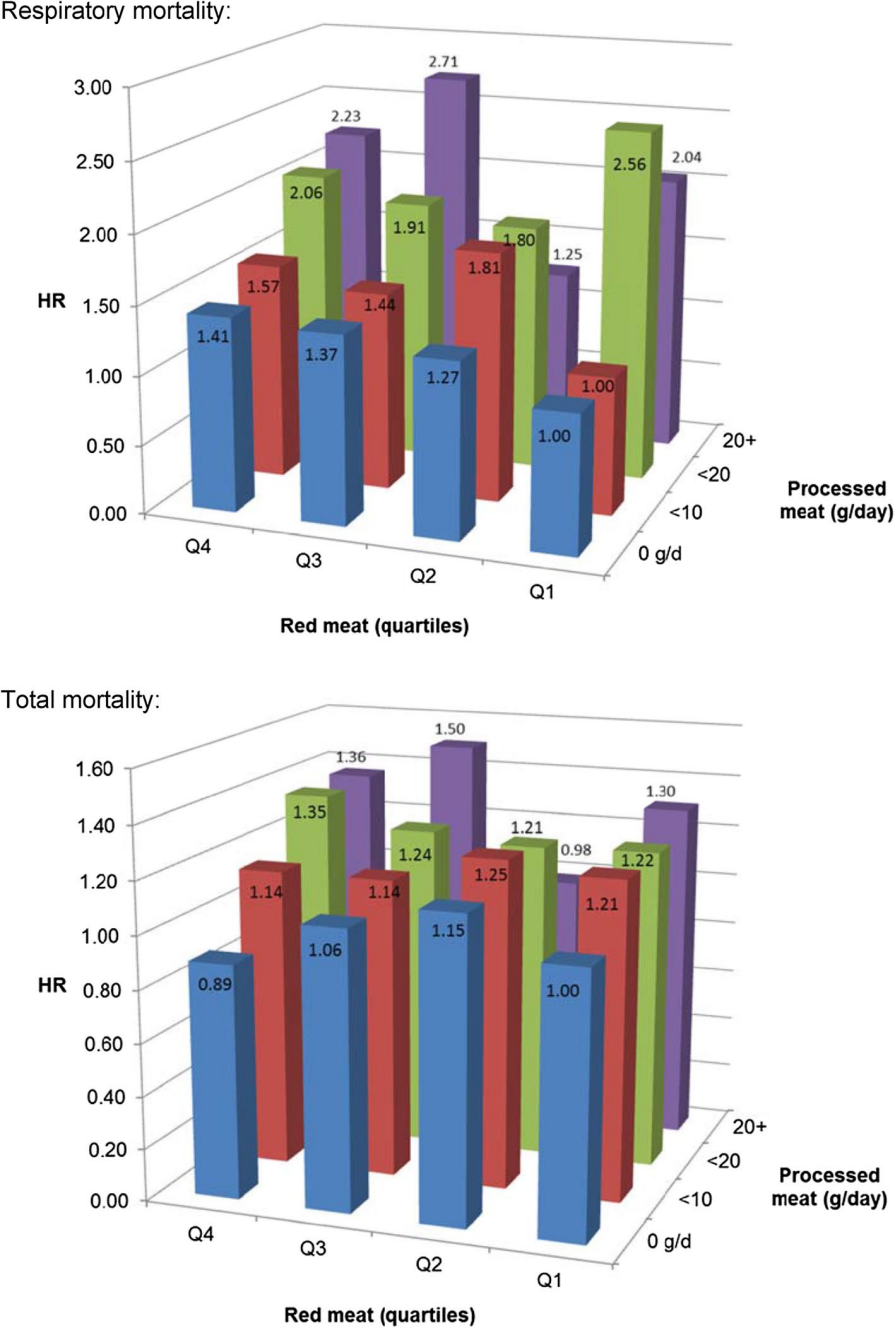
Hazard ratio of respiratory mortality (upper panel) and total mortality (lower panel) according to intake of red meat and processed meat. Multivariable HRs were adjusted for age at baseline (continuous, in years), cigarette smoking status (coded as never, former, current smoker), number of cigarettes smoked per day, and years of smoking (both continuous, centered), history of physician-diagnosed hypertension (no, yes) and diabetes (no, yes), body height (continuous, m), BMI (< 18.5, 18.5 to < 25, 25 to < 30, ≥ 30 kg/m^2^), non-occupational physical activity (< 30, 30–60, 61–90, ≥ 90 min/day), highest level of education (primary school or lower vocational, secondary or medium vocational, and higher vocational or university), intake of alcohol (0, 0.1 to < 5, 5 to < 15, 15 to < 30, 30 + g/day), vegetables and fruit (both continuous, g/day), energy (continuous, kcal/day), use of nutritional supplements (no, yes), and, in women, postmenopausal HRT (never, ever)

Analyses of interaction between red meat and processed meat intake (using a smaller number of categories because of lower numbers in cross-classification) revealed no significant interactions for all mortality endpoints. Figure 4 (upper panel) shows HRs of respiratory mortality for cross-classified quartiles of red meat and categories of processed meat. The figure shows a generally increasing respiratory mortality risk with increasing intake of processed meat, also at low level of red meat intake. For total mortality, Fig. 4 (lower panel) shows this also, and that there is no clear increasing risk with increasing red meat intake.

**Fig. 5:**
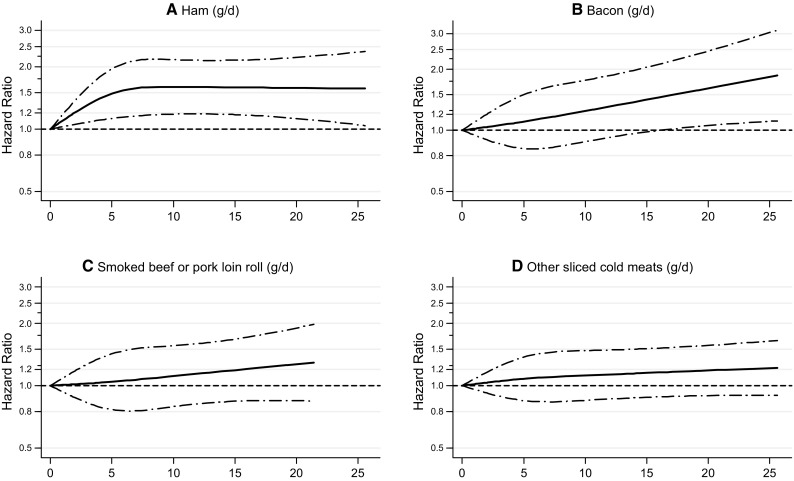
Spline regression curves for the association between types of processed meat intake and respiratory mortality: **a** ham, **b** bacon, **c** smoked beef or pork loin roll, **d** other sliced cold meats. Multivariable HRs were adjusted for age at baseline (continuous, in years), cigarette smoking status (coded as never, former, current smoker), number of cigarettes smoked per day, and years of smoking (both continuous, centered), history of physician-diagnosed hypertension (no, yes) and diabetes (no, yes), body height (continuous, m), BMI (< 18.5, 18.5 to < 25, 25 to < 30, ≥ 30 kg/m^2^), non-occupational physical activity (< 30, 30–60, 61–90, ≥ 90 min/day), highest level of education (primary school or lower vocational, secondary or medium vocational, and higher vocational or university), intake of alcohol (0, 0.1 to < 5, 5 to < 15, 15 to < 30, 30 + g/day), vegetables and fruit (both continuous, g/day), energy (continuous, kcal/day), use of nutritional supplements (no, yes), and, in women, postmenopausal HRT (never, ever)

Because of the relatively high HRs of respiratory mortality associated with processed meat, an additional analysis was conducted regarding individual types of processed meat (Fig. 5). Figure 5 shows that the positive association in particularly present with intake of ham and with bacon, and less so for smoked beef or pork loin roll, or other types of processed meat. (For total mortality, no particular type of processed meat showed a particularly clear positive association, Supplementary Fig S2).

No significant interaction was found between red meat or processed meat intake and smoking status for total, cancer, CVD and respiratory mortality (*P* > 0.2 for all tests). Effect modification analyses with other lifestyle variables revealed no significant interactions (data not shown).

In sensitivity analyses, essentially similar associations with total mortality were seen when analyses limited to stable meat users (Table 2), as well as for cause-specific mortality (data not shown). Analyses excluding the first 2 years of follow-up showed similar results (data not shown). Additional adjustment for heme iron intake only slightly attenuated the associations per 50 g/d increment of red meat with total mortality (HR = 1.01, 95% CI 0.93–1.10) and processed meat with total mortality (HR = 1.09, 95% CI 0.90–1.33). For respiratory mortality, these adjusted associations were (HR = 1.11, 95% CI 0.91–1.34) for red and (HR = 1.56, 95% CI 1.08–2.25) for processed meat, respectively, suggesting only a minor role for heme iron. Conversely, additional adjustment for nitrite intake substantially attenuated the associations of processed meat with total (HR Q5 vs. Q1 = 1.10, 95% CI 0.77–1.55), respiratory (HR = 1.44, 95% CI 0.68–3.05), CVD (HR = 1.09, 95% CI 0.71–1.67), and cancer mortality (HR = 1.11, 95% CI 0.78–1.58). Also, the tests for trend were no longer significant for processed meat. For red meat intake, additional adjustment for nitrite intake did not attenuate the associations with mortality endpoints (data not shown). These observations indicate a possibly important role of nitrite in the associations of processed meat with mortality. Nitrite intake itself was significantly positively associated with total, CVD and respiratory mortality (Supplementary Fig S3).

## Discussion

In this large prospective study, higher processed meat intake was significantly related to higher overall mortality risk in men and women during 10 years of follow-up in men and women aged 55–69 years at baseline, after adjusting for confounders. When comparing subjects in the highest versus lowest intake quintile, the HR for total mortality was 1.21. Significantly positive associations were observed for cardiovascular (HR Q5 vs. Q1, 1.26) and respiratory mortality (HR = 1.79), but not for cancer mortality. Adjustment for nitrite intake considerably attenuated these positive associations, and nitrite was significantly associated with overall, CVD and respiratory mortality. Fresh (unprocessed) red meat intake was not associated with overall and cause-specific mortality. Poultry intake was significantly inversely related to cancer and overall mortality. While fish intake was positively associated with overall and cause-specific mortality, intake of nuts was inversely associated with all endpoints.

Substitution of a combination of poultry, eggs, fish, pulses, nuts and low-fat dairy for processed meat was associated with lower risks of overall, cardiovascular and respiratory mortality.

In other published large cohort studies, red meat intake (unprocessed) was inconsistently associated with total mortality, with some cohort studies showing significantly positive associations [4, 32, 33], but most other studies show no significantly positive association [2, 8, 34, 35], and sometimes even show inverse associations [35]. Processed meat was often significantly positively associated with total mortality, and usually showed stronger positive associations than red meat [2, 33, 34], but not always [4]. Meta-analyses of prospective studies also concluded that processed meat was significantly positively associated, albeit with large heterogeneity, with overall mortality [1, 36] and cancer and cardiovascular mortality [1, 37]. Interestingly, a recent meta-analysis [1] found a significant positive association between unprocessed red meat and total, CVD or cancer mortality only for US cohort studies, but not in European or Asian cohorts [35], although only two European cohort studies were available [2, 3]. The reasons for this difference are unclear, but they could be related to differences in unprocessed red meat intake levels (in US higher than Asia [1, 5, 35]), or cooking practices (more barbecued or grilled in twentieth century in US than in Europe [1]). The median intake of unprocessed red meat in retired AARP-subjects was 63 g/d in men and 49 g/d in women [32], while in the NLCS, median intakes were 89 and 79 g/d in 55–59 year old men and women, respectively. This does not suggest that unprocessed red meat intake in elderly Americans and Dutch differ substantially. Another difference between the US and Europe is the total ban on use of hormonal growth promotors in farm animals in the European Union, while the use of some hormones is authorized under strict conditions in the United States [38]; whether this is related to the observed differences on red meat and mortality remains a question, however.

The NLCS results on red meat and CVD and cancer mortality are in line with those of meta-analyses [1, 37], i.e. no significant association with cancer mortality. In EPIC [2], processed meat was associated with a higher CVD mortality (HR = 1.30 per 50 g/day, 95% CI 1.17–1.45) and cancer mortality. In the NLCS, the corresponding HR for CVD mortality was somewhat lower: 1.24 (1.01–1.53), while no increased cancer mortality was seen. In meta-analyses [1], cancer mortality shows weaker associations with processed meat than CVD mortality, but nevertheless still significant.

Very few studies have looked at possible interaction between red meat and processed meat intake. In Sweden, the combination of unprocessed and processed red meat intake in relation to overall mortality was investigated [34], and they found that unprocessed red meat was not associated with mortality when processed red meat intake was less than 20 g/day. In the NLCS, there was no significant interaction between red meat and processed meat.

While adjustment for heme iron intake did not attenuate the associations with red meat or processed meat importantly in the NLCS, associations between processed meat intake and total, CVD and respiratory mortality were considerably attenuated after additional adjustment for nitrite intake. Since processed meat was the most important source of nitrite in the NLCS [31], these results suggest that nitrite intake may partially explain the associations with processed meat. Processed meat and nitrite were positively associated with total, CVD and respiratory mortality, but showed the strongest associations with respiratory mortality in the NLCS, which is in accordance with findings from AARP cohort [4]. The association between processed meat and respiratory mortality has only been investigated in one other cohort (EPIC) [2], showing a non-significantly positive association. Interestingly, cured meat intake was associated with COPD risk in earlier cohort studies [39, 40], considering that COPD is the main reason for respiratory death and might be linked to N-nitrosamine formation [4, 40].

In the NLCS, we found a moderately strong association between processed meat and respiratory mortality, in particular for ham and bacon intake. Bacon contains relatively high levels of added nitrite, which applies to a lesser extent to ham (pork) [41–44]. Nitrite is traditionally used as food additive in cured meats because of its antimicrobial properties and desirable effects on (red) color and flavor, but there are health concerns related to N-nitrosamine formation. In addition, it has been found that heating of foods, above > 130 °C, may also enhance N-nitrosamine formation. These conditions are favored in cases such as frying bacon, and grilling or frying cured meats or baking pizza [44]. In the late 1970s, high concentrations of N-nitrosamines were detected in fried bacon [45] and also in a Dutch study of 1986 [41], which is close to the 1986 baseline exposure measurement of the NLCS. It is therefore possible that the NLCS has detected associations with nitrite because high levels were used in the past. Permissible amounts of (nitrate and) nitrite in meat curing have been reduced over time in many countries: while maximally 500 mg of nitrite (calculated as NaNO_2_) per kg meat was allowed in curing of meat before 1981, this was reduced to 200 mg/kg in 1981 in The Netherlands [41]. Through EU-legislation, this maximum permitted level was subsequently further lowered in 1995 and 2006 to 150 mg/kg for most meat products [43].

Of course, added salt might also contribute to increased mortality associated with processed meat intake. For both salt and nitrite (and related compounds nitrate and N-nitrosamines), potential mechanisms linking these to CVD mortality are described, involving hypertension and endothelial dysfunction, respectively [46, 47]. Further studies on (types and constituents of) processed meat and particularly respiratory disease and mortality is needed.

While many cohort studies and meta-analyses [13, 14] have shown consistently inverse associations between nut intake and mortality, prospective studies on poultry consumption and mortality show inconsistent results [2, 5, 8, 9, 35]. While poultry intake was significantly inversely related to cancer and overall mortality in the NLCS, it was not related to overall, cancer or CVD mortality in the Golestan cohort [5]. As in the NLCS, poultry intake was significantly inversely related to overall and cancer mortality, but not CVD mortality, in a pooled analysis of Asian cohorts [35]. In Shanghai, poultry showed suggestive inverse association with overall and CVD mortality in men, but not in women [9]. Poultry was not associated with CVD mortality in Japan [48]. In NHANES, poultry consumption tended to be inversely associated with total mortality in men, but not women [8]. In EPIC, poultry was not associated with overall, CVD or cancer mortality [2].

For eggs and legumes (pulses) and mortality, even fewer studies are available [5–7, 49]. While a positive association between egg intake and overall mortality was found in the US Physician’s Health Study [6] and Japan [49], an inverse association was recently reported from the Golestan cohort [5], where egg consumption was considerably lower than in the Japanese study [49]. This also applies to the NLCS, where only subjects consuming < 20 g/d of eggs showed significantly inverse associations with overall mortality compared to nonconsumers, but there was no overall association. As in the NLCS, legume consumption was not related to overall mortality in the Golestan cohort, but there it was inversely related to cancer mortality [5]. In EPIC, legume intake was weakly, borderline significantly associated with an increased overall mortality risk [50]. In a meta-analysis, significant heterogeneity was found according to continent, with an inverse association with legumes only in Asian/Australian studies [15].

Although a recent meta-analysis [12] indicated an inverse association between fish intake and mortality, there was large heterogeneity in the results from different cohort studies in that analysis; inverse associations were generally found in the US and Asian cohorts (except for positive associations with cancer mortality in Asian men [35]), but no or a slightly positive association in European cohorts [15, 18, 19]. This latter observation is in line with the positive associations for overall and cause-specific mortality in the NLCS reported here. In EPIC, no association was found between fish consumption and overall or cause-specific mortality [10]. In Sweden, a U-shaped dose–response association between fish intake and overall mortality was found [17]. Reasons for this difference are unclear, but might be related to fish preparation, where Europeans often fry fish with high fat levels, and to preservation methods, contaminants in fish, and different lifestyles associated with fish consumption.

In line with earlier cohort studies [33], substitution of nuts for processed meat showed the largest contribution to a reduced overall (and cause-specific) mortality risk in the NLCS. While Pan et al. [33] also found significant effects on overall mortality of replacing processed meat with legumes, low-fat dairy, poultry and fish, this was not significant in the NLCS which may be due to a smaller number of deaths. In the NLCS, substitution of eggs or low-fat dairy for processed meat also contributed significantly to a reduced CVD and respiratory mortality risk, as did the combination of poultry, eggs, fish, pulses, nuts and low-fat dairy. This warrants further investigation, because other studies have only looked at substituting total red meat [5].

Strengths of the large-scale NLCS include the prospective design and high completeness of follow-up, which makes information and selection bias unlikely. Apart from the availability of detailed smoking habit data and many other potential confounders for which adjustment was possible, the availability of nitrite and heme iron data in the NLCS enabled further adjustments to investigate whether associations with red meat and processed meat might be attributed to these compounds. The study was also able to look at individual types of processed meat in relation to mortality. Possible reverse causation due to changes in diet or lifestyle was minimized by excluding prevalent CVD or cancer cases [22]. Exclusion of early deaths from follow-up also did not change the results. Sensitivity analyses among stable meat consumers showed comparable associations, reducing the likelihood of reverse causation, although the prior meat consumption question did not distinguish between meat types.

Nevertheless, the NLCS has no updated information on meat intake during follow-up. Because there was no possibility to update dietary or other lifestyle data during follow-up, this may have resulted in some attenuated associations. Other limitations of the NLCS include possible residual confounding, or confounding by unmeasured factors. The validation study of the food frequency questionnaire has shown that it performs relatively well [23], but measurement error may still have attenuated associations. In the NLCS, we do not have exact information about nitrite contents in cured meats 20 years before baseline. In the United States the content of nitrate and nitrite in cured meats decreased by 75% between 1925 and 1981 [51]. Therefore, the intake of nitrite 20 years before baseline is likely to be greater than our estimation of more recent nitrite intake using data from 1984. However, there are no reasons to assume that the categorization (ranking) of individuals in quartiles or quintiles of nitrite intake should be different [52]. Heme–iron content values, as calculated before [30] (i.e. based on type-specific percentage of total iron content) appeared to be in reasonable agreement with absolute heme–iron values in meat and fish available from the same literature sources. Although misclassification can never be ruled out, we have observed positive associations between heme–iron intake and colon cancer risk [30].

In conclusion, this large prospective study suggests that, while red meat intake (unprocessed) is not related to increased mortality, processed meat consumption is related to an increased risk of overall, CVD and respiratory mortality, potentially due to nitrite. Substituting processed meat with other protein sources may lower mortality risk. The findings provide support for public health recommendations to minimize processed meat intake.

## Electronic supplementary material

Below is the link to the electronic supplementary material.

Supplementary material 1 (PDF 105 kb)
