# Supplementary material for: Red meat, processed meat, and other dietary protein sources and risk of overall and cause-specific mortality in The Netherlands Cohort Study
Source: Eur J Epidemiol. 2019 Jan 23;34(4):351–69. doi: 10.1007/s10654-019-00483-9 (PMC6451725; doi:10.1007/s10654-019-00483-9)
Supplement: Supplementary file 1 — Supplementary material 1 (PDF 105 kb) [file 10654_2019_483_MOESM1_ESM.pdf]

SUPPLEMENTARY MATERIALS

European Journal of Epidemiology

Article Title:

Red meat, processed meat, and other dietary protein sources and risk of overall and cause-specific mortality in the Netherlands Cohort Study

Piet A. van den Brandt[1,2]

[1] Maastricht University Medical Centre, GROW- School for Oncology and Developmental Biology, Department of Epidemiology, Maastricht, the Netherlands

[2] Maastricht University Medical Centre, CAPHRI- School for Public Health and Primary Care, Department of Epidemiology, Maastricht, the Netherlands

Address for correspondence:

Piet A. van den Brandt, PhD;

Maastricht University Medical Centre; Department of Epidemiology; PO Box 616; 6200 MD Maastricht; The Netherlands; Tel.: +31 (0)43 3882361; E-mail:

PA.vandenBrandt@maastrichtuniversity.nl

Supplementary Table 1. Baseline characteristics (means, or percent) of deceased subjects and subcohort members with complete dietary and covariable data, Netherlands Cohort Study 1986-1996.

Characteristic	Men		Women	
	Total deaths	Subcohort	Total deaths	Subcohort
N	5797	1535	3026	1667
Age, mean (yr)	63.1	61.3	63.2	61.5
BMI (kg/m^2)	24.9	24.9	25.1	25.0
Physical activity, nonoccupational (minutes/day)	75.9	80.7	58.5	66.3
Alcohol intake (g/day)	16.7	15.1	6.0	6.1
Vegetable intake (g/day)	180.6	186.5	183.3	193.0
Fruit intake (g/day)	145.5	155.9	186.5	195.5
Red meat intake (g/day)	94.8	94.0	80.6	80.8
Processed meat intake (g/day)	16.3	16.3	10.8	10.5
Poultry intake (g/day)	12.6	13.2	12.6	13.1
Eggs intake (g/day)	17.4	17.1	15.1	14.8
Fish intake (g/day)	15.1	13.9	11.6	11.3
Pulses intake (g/day)	9.4	9.5	6.6	6.8
Nut intake (g/day)	6.4	8.3	3.7	4.5
Low-fat dairy (g/day)	168.0	177.8	194.1	200.8
Never smoker (%)	9.7	14.5	54.1	59.0
University or higher vocational education (%)	17.7	21.4	7.8	10.3
Diabetes (%)	5.4	2.9	7.9	3.0
Hypertension (%)	26.3	22.4	36.1	27.2
Nutritional supplement user (%)	22.1	23.5	34.1	37.7
Ever used hormone replacement therapy (%)			10.9	13.3

Supplementary Figure S1. Flow diagram of the number of subcohort members and deaths on which analyses are based, Netherlands Cohort Study.

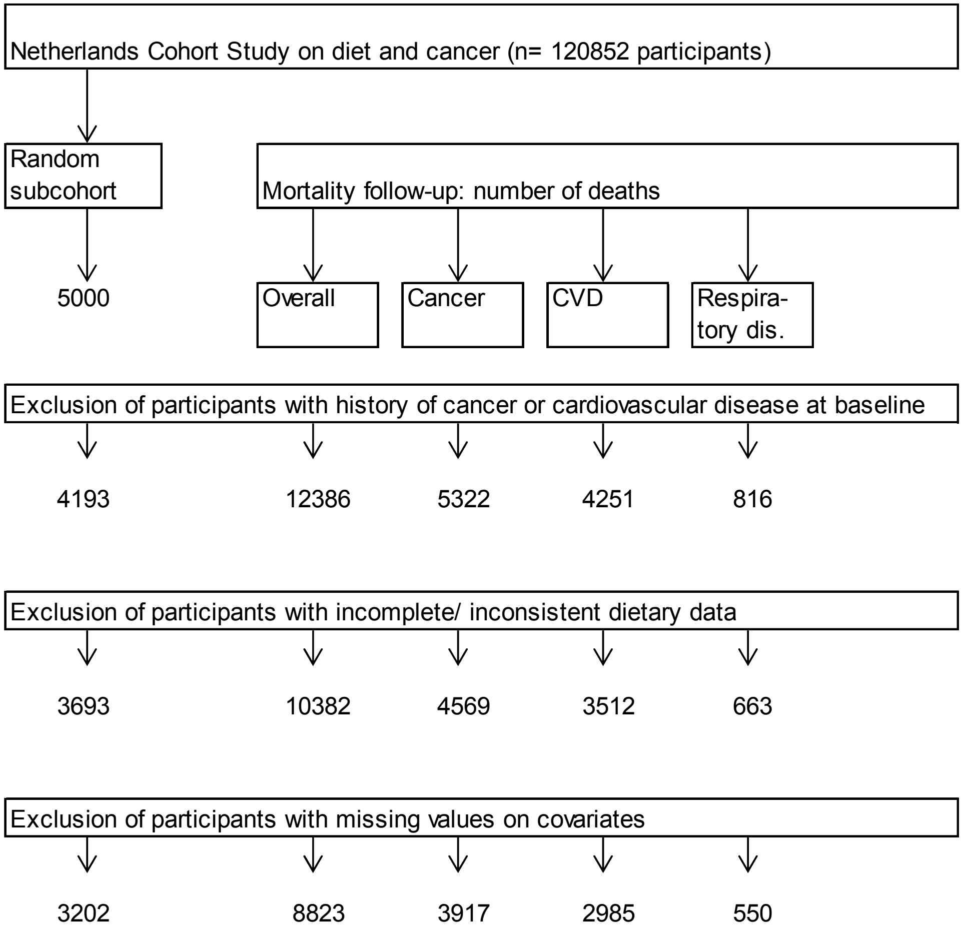

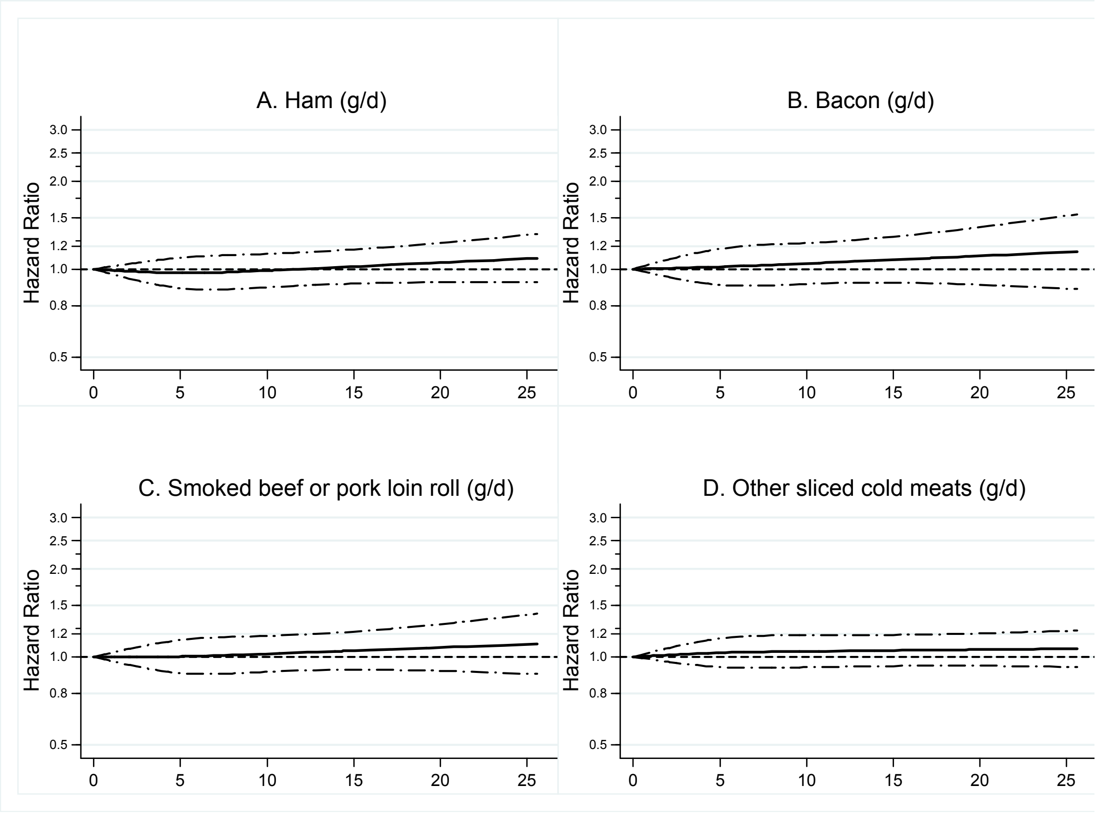

Supplementary Fig S2. Spline regression curves for the association between types of processed meat intake and total mortality: A) ham, B) bacon, C) smoked beef or pork loin roll and D) other sliced cold meats.

Multivariable HRs were adjusted for age at baseline (continuous, in years), cigarette smoking status (coded as never, former, current smoker), number of cigarettes smoked per day, and years of smoking (both continuous, centered), history of physician-diagnosed hypertension (no, yes) and diabetes (no, yes), body height (continuous, m), BMI (<18.5, 18.5-<25, 25-<30, ≥30 kg/m^2), non-occupational physical activity (<30, 30-60, 61-90, ≥90 min/day), highest level of education (primary school or lower vocational, secondary or medium vocational, and higher vocational or university), intake of alcohol (0, 0.1-<5, 5-<15, 15-<30, 30+ g/day), vegetables and fruit (both continuous, g/day), energy (continuous, kcal/day), use of nutritional supplements (no, yes), and, in women, postmenopausal HRT (never, ever).

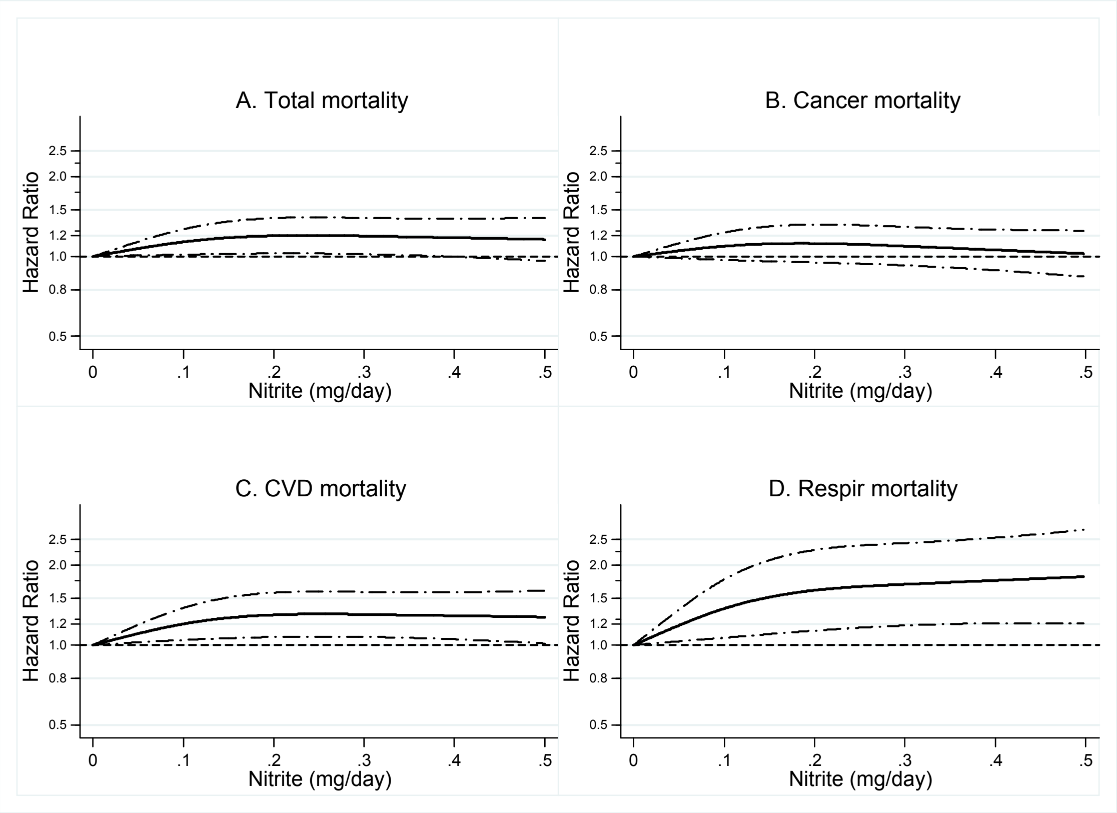

Supplementary Fig S3. Spline regression curves for the association between nitrite intake and A) total mortality, B) cancer mortality, C) CVD mortality and D) respiratory mortality. Multivariable HRs were calculated by restricted cubic spline regression (using 3 knots at 10th, 50th, and 90th percentiles) adjusting for: age at baseline (continuous, in years), cigarette smoking status (coded as never, former, current smoker), number of cigarettes smoked per day, and years of smoking (both continuous, centered), history of physician-diagnosed hypertension (no, yes) and diabetes (no, yes), body height (continuous, m), BMI (<18.5, 18.5-<25, 25-<30, ≥30 kg/m^2), non-occupational physical activity (<30, 30-60, 61-90, ≥90 min/day), highest level of education (primary school or lower vocational, secondary or medium vocational, and higher vocational or university), intake of alcohol (0, 0.1-<5, 5-<15, 15-<30, 30+ g/day), vegetables and fruit (both continuous, g/day), energy (continuous, kcal/day), use of nutritional supplements (no, yes), and, in women, postmenopausal HRT (never, ever).
